# Tumor–Stroma Cross-Talk in Human Pancreatic Ductal Adenocarcinoma: A Focus on the Effect of the Extracellular Matrix on Tumor Cell Phenotype and Invasive Potential

**DOI:** 10.3390/cells7100158

**Published:** 2018-10-05

**Authors:** Patrizia Procacci, Claudia Moscheni, Patrizia Sartori, Michele Sommariva, Nicoletta Gagliano

**Affiliations:** 1 Department of Biomedical Sciences for Health, Università degli Studi di Milano, via L. Mangiagalli 31, 20133 Milan, Italy; patrizia.procacci@unimi.it (P.P.); patrizia.sartori@unimi.it (P.S.); michele.sommariva@unimi.it (M.S.); 2 Department of Biomedical and Clinical Sciences “L. Sacco”, Università degli Studi di Milano, via G.B. Grassi 74, 20157 Milan, Italy; claudia.moscheni@unimi.it

**Keywords:** epithelial-to-mesenchymal transition, E-cadherin, MMPs, cell migration, extracellular matrix remodeling

## Abstract

The extracellular matrix (ECM) in the tumor microenvironment modulates the cancer cell phenotype, especially in pancreatic ductal adenocarcinoma (PDAC), a tumor characterized by an intense desmoplastic reaction. Because the epithelial-to-mesenchymal transition (EMT), a process that provides cancer cells with a metastatic phenotype, plays an important role in PDAC progression, the authors aimed to explore in vitro the interactions between human PDAC cells and ECM components of the PDAC microenvironment, focusing on the expression of EMT markers and matrix metalloproteinases (MMPs) that are able to digest the basement membrane during tumor invasion. EMT markers and the invasive potential of HPAF-II, HPAC, and PL45 cells grown on different ECM substrates (fibronectin, laminin, and collagen) were analyzed. While N-cadherin, αSMA, and type I collagen were not significantly affected by ECM components, the E-cadherin/β-catenin complex was highly expressed in all the experimental conditions, and E-cadherin was upregulated by collagen in PL45 cells. Cell migration was unaffected by fibronectin and delayed by laminin. In contrast, collagen significantly stimulated cell migration and the secretion of MMPs. This study’s results showed that ECM components impacted cell migration and invasive potential differently. Collagen exerted a more evident effect, providing new insights into the understanding of the intricate interplay between ECM molecules and cancer cells, in order to find novel therapeutic targets for PDAC treatment.

## 1. Introduction

Pancreatic ductal adenocarcinoma (PDAC) is one of the most aggressive carcinomas, characterized by a dismal prognosis due to the high incidence of recurrence and metastases dissemination. PDAC is the fourth leading cause of cancer-related mortality in the Western world, with an estimated incidence of more than 40,000 cases per year in the United States. Due to the high recurrence and malignancy, the overall five-year survival rate for all stages of the disease is <7% [1,2,3].

During PDAC progression, the pancreatic tumor microenvironment (TME) containing extracellular matrix (ECM) components, growth factors, and soluble mediators, and different non-parenchymal stromal cells including fibroblasts, inflammatory, and pancreatic stellate cells, undergoes evident qualitative and quantitative modifications. In addition, it plays a key role as a modulator of cancer cell phenotype, behavior, and chemoresistance [4,5].

PDAC is characterized by an intense “desmoplastic reaction”, defined as the host fibrotic response to the invasive carcinoma, consisting of the abnormal accumulation of ECM components, mostly collagen fibers [6]. The desmoplastic reaction represents the histological hallmark of PDAC, often accounting for 50–80% of the tumor volume [4,7]. Desmoplasia allows for a complex and dynamic interplay among invading tumor cells, normal host epithelial cells, fibroblasts, ECM components, and released cytokines, growth, and angiogenic factors [8]. ECM acts as a physical scaffold, facilitating interactions between different cell types, providing survival and differentiation signals, and affecting resistance to anticancer drugs. Therefore, ECM represents an important mediator of cancer cell behavior, influencing tumor cell proliferation and migration [9], as well as tissue homeostasis.

Key molecules occurring in the pancreatic stroma and the desmoplastic reaction have been identified, such as collagen types I, IV, and V, and fibronectin, laminin, matrix metalloproteinases (MMPs) and their inhibitors, tissue inhibitors of metalloproteinases (TIMPs), and transforming growth factor-β1 (TGF-β1) [10].

Among ECM molecules, type I collagen (COL-I) is the most abundant in pancreatic TME. Previous studies suggested that it was associated with increased integrin-mediated cell–cell adhesion, proliferation, and the migration of PDAC cells [11]. Basement membrane collagen type IV and laminin provide a proper microenvironment for pancreatic cancer cells affecting the cytotoxicity of anticancer drugs, and favoring cancer cell growth [9]. In contrast, the role of type V collagen, a minor component of ECM, is still poorly understood, since it triggers opposite cellular responses depending on the cell type. For instance, in breast cancer type V, collagen promotes breast cancer cell apoptosis [12], and its decrease in lung cancer is associated with increased tumor growth rate, motility, and invasion, as well as increased angiogenesis [13]. Fibronectin is abundant in both chronic pancreatitis and pancreatic cancer, suggesting that this protein may favor the development of pancreatic cancer [14].

MMPs, also considered as markers of epithelial-to-mesenchymal transition (EMT) (especially MMP-2 and MMP-9), render tumor cells able to metastasize in distant organs by breaking down the basement membrane, thus allowing cancer cells to enter the lymphatic or blood vessels [15]. The phenotype of carcinoma cells undergoing EMT is characterized by the loss of epithelial characteristics, especially the down-regulation of E-cadherin, leading to the loss of cell adhesion and polarity. Cytoskeleton reorganization, vimentin, and α-smooth muscle actin (αSMA) expression are also observed, as well as an increased degree of motility and secretion by MMPs [16].

Considering the role of desmoplasia in PDAC, the authors aimed to investigate the effects of single ECM components that are present in the TME on the PDAC cell phenotype, focusing on EMT pathways and MMP expression, in order to better understand the intricate cell–ECM cross-talk.

## 2. Materials and Methods

### 2.1. Cell Cultures

Three human pancreatic cancer cell lines (HPAF-II, HPAC, and PL45) from pancreatic ductal adenocarcinoma (PDAC) (American Type Culture Collection, ATCC, Manassas, VA, USA) were studied. PDAC cells were cultured in Dulbecco’s Modified Eagle’s Medium (DMEM) supplemented with 10% heat-inactivated fetal bovine serum (FBS), 2 mM glutamine, antibiotics (100 U/mL penicillin, 0.1 mg/mL streptomycin), and 0.25 μg/mL amphotericin B. Cell viability was determined by Trypan blue staining.

Cells were cultured in duplicate on Petri dishes coated with fibronectin (FN), laminin (LAM), COL-I (COL) (Cellcoat, Greiner Bio-One, Cassina de Pecchi-Milan, Italy), or without coating (NC), in order to characterize the specific effect of these proteins on PDAC cell phenotype.

### 2.2. Immunofluorescence

The expression of key EMT markers and their localization were assessed by immunofluorescence in PDAC cells grown on 12 mm-diameter rounded coverslips coated with FN, LAM, COL-I (Neuvitro Corporation, Vancouver, WA, USA), or uncoated. When they were at the desired confluence, cells were washed in phosphate-buffered saline (PBS), fixed in 4% paraformaldehyde in PBS-containing 2% sucrose for 10 min at room temperature, post-fixed in 70% ethanol, and stored at −20 °C until use. Cells were incubated with the primary antibodies anti-E-cadherin (1:2500, Becton Dickinson, Milan, Italy), anti β-catenin (1:500, Novocastra, Newcastle upon Tyne, UK), anti-N-cadherin (1:200, Santa Cruz Biotechnology, Heidelberg, Germany), anti-COL-I (1:2000, Sigma-Aldrich, Milan, Italy), anti-vimentin (1:200, Novocastra), and anti-αSMA (1:400, Sigma-Aldrich). Secondary antibodies conjugated with Alexa 488 (1:500, Molecular Probes, Invitrogen, Waltham, MA, USA) were applied for 1 h at room temperature in PBS. Negative controls were incubated omitting the primary antibody. Finally, after incubation with 4′,6-Diamidine-2′-phenylindole dihydrochloride (DAPI) (1:100,000, Sigma-Aldrich), the coverslips were mounted on glass slides using Mowiol.

### 2.3. Western Blot

Cell lysates were prepared in Tris-HCl 50 mM pH 7.6, 150 mM NaCl, 1% Triton X-100, 5 mM ethylenediaminetetraacetic acid (EDTA), 1% sodium dodecyl sulphate (SDS), proteases inhibitors, and 1 mM sodium orthovanadate. Lysates were incubated on ice for 30 min and centrifuged at 14,000 *g* for 10 min at 4 °C to remove cell debris. Cell lysates (40 µg of total proteins) were diluted in SDS sample buffer, loaded on 10% SDS polyacrylamide gel, separated under reducing and denaturing conditions at 80 V according to Laemmli, and transferred at 90 V for 90 min to a nitrocellulose membrane in 0.025 M Tris, 192 mM glycine, and 20% methanol, pH 8.3. For E-cadherin evaluation, membranes were incubated for 1 h at room temperature with monoclonal antibodies to E-cadherin (1:2500, Becton Dickinson) and, after washing, in horseradish peroxidase (HRP)-conjugated rabbit anti-mouse serum (1:40,000 dilution, Sigma-Aldrich). To confirm equal loading, membranes were reprobed by monoclonal antibody to α-tubulin (1:2000 dilution, Sigma-Aldrich). Immunoreactive bands were revealed using the Amplified Opti-4CN (Bio Rad, Hercules, CA, USA).

### 2.4. SDS-Zymography

Serum-free culture media were mixed 3:1 with sample buffer (containing 10% SDS). Samples (5 μg of total protein per sample) were run under non-reducing conditions without heat denaturation on 10% polyacrylamide gel (SDS-PAGE) co-polymerized with 1 mg/mL of type I gelatin. The gels were run at 4°C. After SDS-PAGE, the gels were washed twice in 2.5% Triton X-100 for 30 min each, and incubated overnight in a substrate buffer at 37 °C (Tris-HCl 50 mM, CaCl_2_ 5 mM, 0.02% NaN_3_, pH 7.5). MMP gelatinolytic activity, detected after staining the gels with Coomassie brilliant blue R250 as clear bands on a blue background, was quantified by densitometric scanning (UVBand, Eppendorf, Milan, Italy).

### 2.5. Wound Healing Assay

The cell migration of PDAC cells was analyzed by wound healing assay [17] in confluent cells using 2-well silicone culture-inserts (Ibidi, Martinsried, Germany) in Petri dishes coated with FN, LAM, COL-I, or uncoated (NC). After removal of the insert, migration of cells was assessed by measuring the closure of the wound at different time points. Petri dishes were incubated at 37 °C and observed under an inverted microscope at different time points. Digital images were captured by a digital camera after 0 and 27 h, and the size of the “scratch” was measured to obtain the migration potential. 

### 2.6. Statistical Analysis

Statistical analysis was performed using GraphPad Prism software (GraphPad Software Inc., version 6.0, La Jolla, CA, USA). Data were obtained from two replicate experiments for each cell line in each experimental condition cultured in duplicate and were expressed as mean ± standard deviation (SD). Comparison of groups was calculated using one-way ANOVA. Differences associated with *p*-values lower than 5% were to be considered significant.

## 3. Results

### 3.1. Cell Morphology is not Influenced by ECM Components

**Figure 1:**
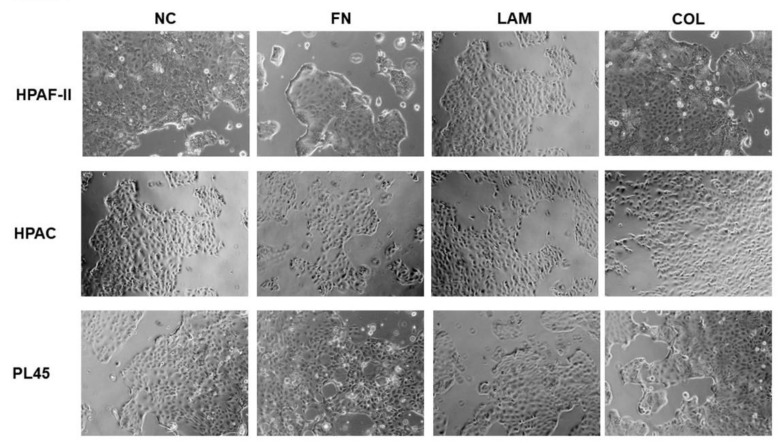
Cell morphology. Photomicrographs at the inverted microscope showing the epithelial morphology of HPAF-II, HPAC, and PL45 cells grown on fibronectin (FN), laminin (LAM), COL-I (COL), or without coating (NC) (original magnification 10×).

HPAF-II, HPAC, and PL45 cell lines, normally characterized by an epithelial phenotype, retained their morphology after culture on FN, LAM, COL-I, or without coating (NC) as demonstrated by the observation at the inverted microscope (see Figure 1), suggesting that these three molecules do not alter cell conformation.

### 3.2. EMT Markers Are Differently Expressed in Cells Grown on Different ECM Components

**Figure 2:**
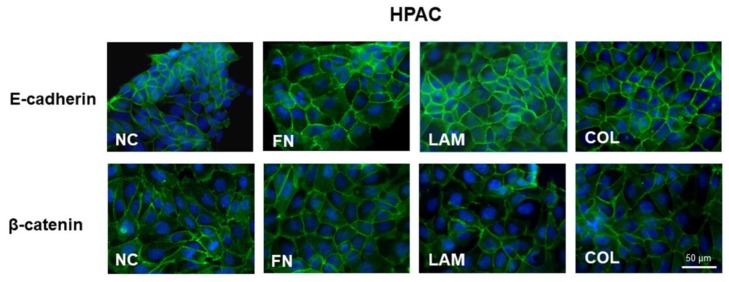
Immunofluorescence analysis of epithelial markers. Photomicrographs showing the expression of the epithelial markers E-cadherin and β-catenin in HPAC cells cultured on FN, LAM, COL, or without coating (NC) (original magnification 60×).

Immunofluorescence analysis revealed that E-cadherin is strongly expressed at cell boundaries in HPAC cells cultured on FN, LAM, COL-I, or without coating (NC), suggesting the presence of functional adherens junctions (see Figure 2). A similar pattern was observed for β-catenin immunoreactivity, detectable at the plasma membrane in all experimental conditions. These results indicated that the E-cadherin/β-catenin complex was not modified by the considered ECM components (see Figure 2). The same immunoreactivity was observed in HPAF-II and PL45 cells (data not shown).

**Figure 3:**
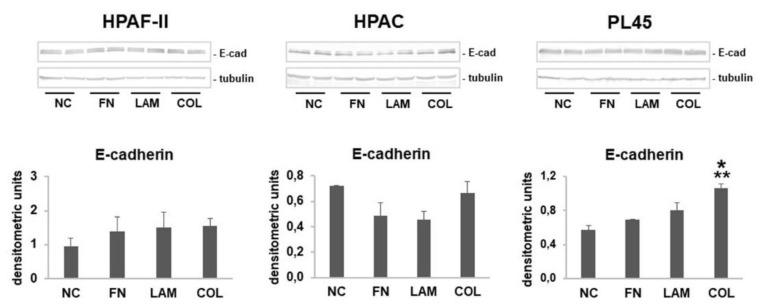
E-cadherin protein levels. Representative Western blot analysis and bar graphs showing E-cadherin expression in whole cell lysates of HPAF-II, HPAC, and PL45 cells cultured on FN, LAM, COL, or without coating (NC). Data are means ± SD. * *p* < 0.01 vs. NC; ** *p* < 0.05 vs. FN, LAM.

Since immunofluorescence does not allow a precise quantification of protein expression, E-cadherin protein levels were quantified by Western blot. According to immunofluorescence analysis, E-cadherin expression was not significantly modulated in HPAF-II and HPAC cells cultured on FN, LAM, COL-I, or without coating (NC). In contrast, it was strongly induced in PL45 cells that were grown on COL compared to NC controls (*p* < 0.01 vs. NC, *p* < 0.05 vs. FN, LAM) (see Figure 3).

**Figure 4:**
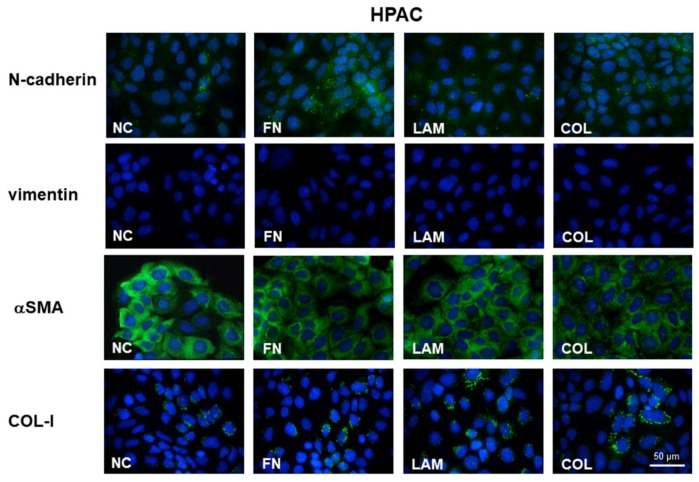
Immunofluorescence analysis of mesenchymal markers. Photomicrographs showing the expression of mesenchymal markers N-cadherin, vimentin, αSMA, and COL-I in HPAC cells cultured on FN, LAM, COL, or without coating (NC) (original magnification 60×).

The analysis of mesenchymal markers in HPAC cells showed that N-cadherin was expressed at very low levels in HPAC cells, although this seemed to be slightly more evident in the cytoplasm of some cells that were cultured on FN and COL-I (see Figure 4). Vimentin was undetectable under all experimental conditions, whereas αSMA was highly expressed in all PDAC cells under the different experimental conditions (see Figure 4). COL-I immunoreactivity was detectable in cells cultured on all the substrates, but immunoreactivity seemed more evident in cells that were grown on LAM and COL (see Figure 4).

### 3.3. Cell Migration is Differently Stimulated by ECM Components

**Figure 5:**
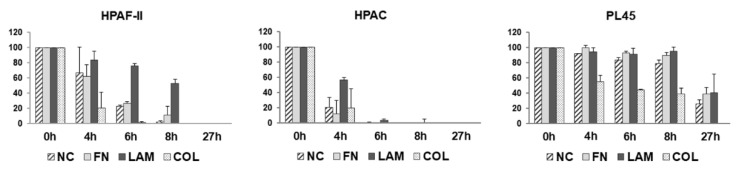
Cell migration. Bar graphs showing the closure of the wound at the indicated time points in HPAF-II, HPAC, and PL45 cells cultured on FN, LAM, COL, or without coating (NC). Bars represent the open wound, and data are expressed as the % vs. time point 0 h. Data are mean ± SD.

The data of cell migration assessed by a wound healing assay at the considered time points are shown in Figure 5. Although migration appeared slightly reduced in HPAF-II cells, a similar trend was observed in HPAF-II and HPAC cells cultured on FN (38%, 73%, 89%, and 100% closure for HPAF-II after, respectively, 4 h, 6 h, 8 h, and 27 h; 88%, 100%, 100%, and 100% closure after for HPAC, respectively, 4 h, 6 h, 8 h, and 27 h). A different pattern was observed for LAM. In fact, migration was very slow in both HPAF-II and HPAC cells, compared not only to NC, but also to FN and COL. In contrast, PL45 exhibited a slower migration, compared to HPAF-II and HPAC cells, and in these cells, migration was similar at all the considered time points for cells grown on FN, as well as on LAM. When analyzing the effect of COL, increased migration was evident, especially in HPAF-II and HPAC cells (79%, 99%, 100%, and 100% closure after, respectively, 4 h, 6 h, 8 h, and 27 h for HPAF-II; 80%, 100%, 100%, and 100% closure after, respectively, 4 h, 6 h, 8 h, and 27 h for HPAC cells). For PL45, although at a lower extent, migration was strongly induced by COL, compared to NC, FN, and LAM (45%, 56%, 60%, and 100% closure after, respectively, 4 h, 6 h, 8 h, and 27 h for PL45 cells).

### 3.4. MMP Levels and Activity Are Affected by ECM Components

**Figure 6:**
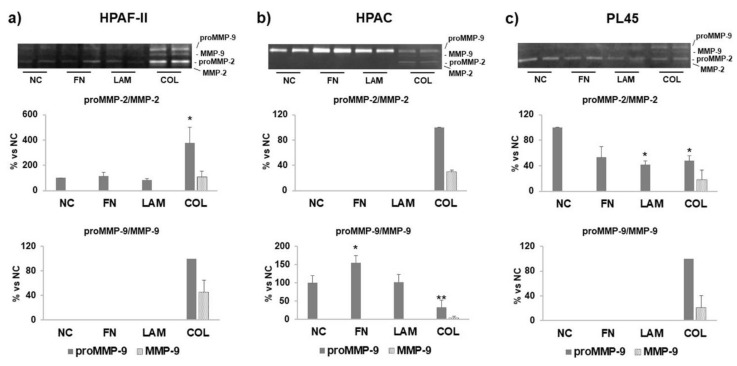
Matrix metalloprotenases-2 (MMP-2) and -9 (MMP-9) activity. Representative gelatin zymograms and bar graphs showing MMP-2 and MMP-9 activity in supernatants from (**a**) HPAF-II, (**b**) HPAC, and (**c**) PL45 cells cultured on FN, LAM, COL, or without coating (NC). Matrix metalloproteinases (MMPs) activity was induced, especially by COL. Data are expressed as % vs. NC and are means ± SD. (**a**) * *p* < 0.001 vs. NC, FN, LAM; (**b**) * *p* < 0.05 vs. NC, LAM; ** *p* < 0.01 vs. NC, FN, LAM; (**c**) * *p* < 0.05 vs. NC.

The invasive potential of PDAC cells was assayed by SDS-zymography. The results show that HPAF-II and PL45 cells expressed mostly MMP-2, whereas HPAC cells were characterized by a high expression of MMP-9. Both MMP-2 and MMP-9 were similarly expressed in HPAF-II cells cultured on FN, LAM, or without coating (NC), but they were significantly induced by COL-I (*p* < 0.001 for proMMP-2 vs. NC, FN, LAM) (see Figure 6a). A similar pattern was observed in HPAC for MMP-2, whereas MMP-9 was significantly induced when HPAC cells were cultured on FN (*p* < 0.05 vs. NC, LAM) (see Figure 6b). In PL45, MMP-2 activity was significantly reduced by LAM (*p* < 0.05 vs. NC), as well as by COL (*p* < 0.05 vs. NC). However, COL-I induced the expression of the active MMP-2 and a strong increase of MMP-9 (see Figure 6c). Collectively, MMP secretion resulted in a strong induction in all three cell lines cultured on COL-I. Moreover, in cells grown on COL-I, the active forms of MMPs were detectable.

## 4. Discussion

PDAC is characterized by an intense desmoplastic reaction, a fibrotic lesion determined by an abnormal accumulation of ECM components, especially COL-I [4]. Interestingly, it has been demonstrated that primary tumors and metastatic lesions exhibited similar levels of desmoplasia, including high levels of some ECM components such as COL-I, COL-III, and COL-IV [18], and the expression of markers of desmoplasia has also been detected in metastatic sites [19]. Therefore, metastatic lesions are also fibrotic, like primary tumors, thus suggesting a key role for ECM components in the desmoplastic reaction of PDAC.

ECM is a dynamic structure, acting also as a physical scaffold, because the interaction between cells and the ECM through integrins or other cell surface receptors triggers intracellular signaling pathways that can influence cell survival, differentiation, chemoresistance, angiogenesis proliferation, migration, and invasion, which are all processes that contribute to cancer progression [20,21]. COL-I is the most abundant component of ECM in the tumor stroma, and it is highly expressed in metastatic tumors [22,23]. Previous studies investigated the effect of COL-I on pancreatic carcinoma cells, showing that high levels of COL-I significantly correlated with a reduced overall survival of PDAC patients [18], and influenced E-cadherin expression [24,25].

In this study, the authors characterized the effect of single ECM components on the PDAC cell phenotype, focusing their attention especially on E-cadherin and MMP expression. E-cadherin down-regulation is considered to be a key event during EMT, as demonstrated in vivo and in different cancer cell lines, including lung, breast, colorectal, and ovarian cancer [26,27,28]. The so-called “cadherin switch”, a reduced expression of E-cadherin paralleled with an increased expression of N-cadherin, was described in PDAC [29,30]. Although E-cadherin down-regulation is considered an early essential event in EMT, experimental evidence demonstrates that six out of seven PDAC commercial cell lines maintain E-cadherin expression on the cell membrane [31], supporting the importance of studies investigating the role of E-cadherin, and more generally of EMT markers, in PDAC progression.

In this study, the authors showed that E-cadherin was strongly expressed in PDAC cells cultured on the different substrates and was significantly upregulated in PL45 grown on COL-I, suggesting that this ECM component elicited an important effect on tumor cells. According to their previous study [32], the maintenance of E-cadherin at cell–cell boundaries in PDAC cells showed that adherens junctions, and therefore cell adhesion, were preserved. This phenotypic characteristic of PDAC cells was needed to ensure tissue integrity during collective cell migration [33], and this study’s results suggested that COL-I in the tumor stroma could contribute to PDAC malignant behavior.

ECM in the tumor stroma is a dynamic structure and stromal ECM undergoes a finely regulated dynamic turnover mediated by MMP enzymatic activity [15]. ECM degradation allows the migration of invasive cells into the surrounding tissue and vasculature [34]. The authors investigated the effect of ECM components on MMP-2 and MMP-9, because they are the key effectors that are involved in the degradation of the basement membrane, an important step during tumor invasion. Moreover, the roles of MMP-2 and MMP-9 in metastasis in pancreatic cancer are also demonstrated by a positive correlation between the expression of these two MMPs and the microvessel density, suggesting their involvement in the angiogenic processes [35]. In pancreatic cancer, MMP-2 is produced and secreted by both tumor and stromal cells [36], and a strong correlation between MMP-2 expression and the invasive potential of pancreatic cell lines has been observed [37]. MMP-9 expression is associated with lymph node invasion and the occurrence of distant metastases. Moreover, a correlation between MMP-9 expression and a worse prognosis in PDAC patients was found [38]. Previous studies indicated that MMP-9 production was influenced by the TME [39], suggesting that the stroma may act as a key facilitator of tumor invasion.

This study’s results showed that PDAC cells had a different MMP secretion profile and activity, because HPAF-II and PL45 cells expressed mostly MMP-2, whereas HPAC cells expressed mostly MMP-9. Independently of the MMP expressed, the authors observed a different effect of ECM components on the PDAC cells considered. COL-I strongly induced MMP-2 and MMP-9 activity in HPAF-II cells, MMP-2 in HPAC, and MMP-9 in PL45, pointing to a pivotal influence of COL-I on the invasive potential of PDAC cells. These data are consistent with the wound healing assay results, showing an increased migration of cells cultured on COL-I. Because migration and invasive potential are both necessary to allow tumor invasion, and are both strongly stimulated by COL-I, the authors can hypothesize that COL-I is the component of the ECM that plays a pivotal role in influencing tumor cell behavior. MMP levels were differently affected by FN and LAM, depending on cell type, leading to the hypothesis that the effect of ECM components is dependent on tumor cell phenotype, and therefore, on the differentiation grade. However, a relationship between differentiation grade, cell migration, and invasion potential has not yet been clearly defined [40].

**Figure 7:**
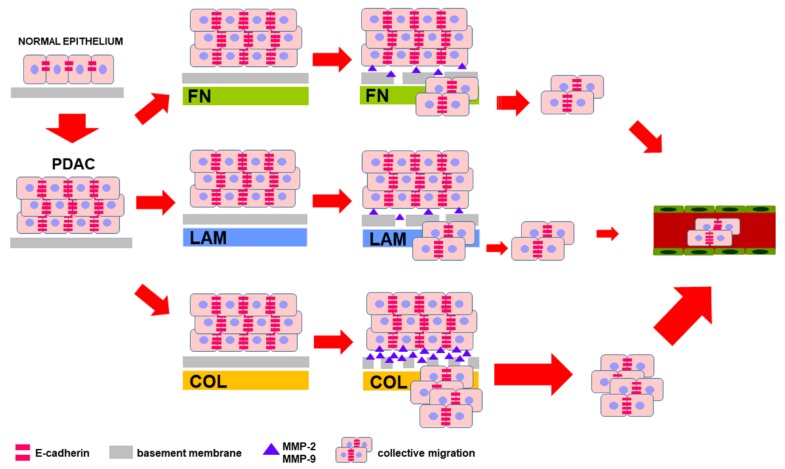
Extracellular matrix (ECM) components differentially affect pancreatic ductal adenocarcinoma (PDAC) cell migration and invasive properties. Diagram summarizing the hypothesis based on the most striking characteristics of PDAC cells grown on different ECM components used as a substrate. High-level expression of E-cadherin at cell boundaries is preserved and allows collective cell migration and invasion, favored by COL occurring in the pancreatic tumor microenvironment (TME), which stimulates both migration and secretion of MMPs.

This study’s findings, summarized in Figure 7, point to COL-I as a key influencer of PDAC cell phenotype and behavior. In PDAC stroma, COL-I is secreted by pancreatic stellate cells and myofibroblasts, acting as a crucial player in the development and maintenance of desmoplasia [4]. In this study, the authors showed that ECM components stimulated COL-I expression in PDAC cells, which very likely contributed to the secretion and deposition of COL-I in the tumor microenvironment.

## 5. Conclusions

Collectively, the authors’ results are consistent with the pivotal role of desmoplasia and ECM components, occurring in the PDAC, in influencing tumor cell behavior, especially migration and invasive potential. This is achieved possibly by triggering signaling pathways and affecting anti-tumor drug penetration through the tumor. Although targeted therapies directed against stroma components have appeared to be an appealing therapeutic approach for the treatment of PDAC [41], recent studies have demonstrated that the depletion of the stroma [42] or of activated myofibroblasts [43] elicited a paradoxical result, rendering the tumor more aggressive. Moreover, it was suggested that the final tumor-promoting or tumor-suppressive effect of stroma components depended on the differentiation grade of cancer cells [42], further demonstrating the complexity of the microenvironment and of the bidirectional and mutual tumor–cell cross-talk. More recently, it was suggested that pharmacological stromal “normalization”, used in order to achieve the homeostatic restoration of desmoplastic stroma, may represent a novel and promising therapeutic approach for PDAC [44]. In this complex context, the authors’ data contribute to the understanding of the intricate cellular interactions between stromal ECM components and cancer cells, and stimulate further research on the ECM components in PDAC progression, in order to find more effective therapeutic tools for PDAC treatment.
